# Local gingival crevicular fluid, synovial fluid, and circulating levels of prolactin hormone in patients with moderately active rheumatoid arthritis and stage III and IV periodontitis before and after non-surgical periodontal treatment—a controlled trial

**DOI:** 10.1007/s00784-023-04867-w

**Published:** 2023-01-31

**Authors:** Naglaa Mohamed El-Wakeel, Zienab Farid Shalaby, Rania Farouk Abdulmaguid, Sally Said Abd Elhamed, Olfat Shaker

**Affiliations:** 1 Oral Medicine and Periodontology Department, Faculty of Dentistry, Al-Azhar University (Girls Branch), Al-Mokhayam El Dayem St., Nasr City, 1178 Cairo Egypt; 2 Oral Medicine and Periodontology Department, Faculty of Dentistry, October University for Modern Sciences and Arts, Giza, Egypt; 3 Internal Medicine Department, Faculty of Medicine, Al-Azhar University (Girls Branch), Cairo, Egypt; 4 Medical Biochemistry and Molecular Biology Department, Faculty of Medicine, Cairo University, Cairo, Egypt

**Keywords:** Gingival crevicular fluid, Periodontitis, Prolactin, Rheumatoid arthritis, Synovial fluid

## Abstract

**Objectives:**

We aimed to investigate prolactin (PRL) levels in gingival crevicular fluid (GCF), synovial fluid, and serum in patients suffering from moderately active rheumatoid arthritis (RA) with and without periodontitis (P). Further, to evaluate the effect of non-surgical periodontal treatment on these levels compared to controls.

**Materials and methods:**

Eighty subjects were divided into 4 groups: group 1: 20 patients with RA + P, group 2: 20 periodontitis patients (systemically healthy), group 3: RA patients (periodontally healthy), and group 4: healthy controls. Patients with periodontitis received scaling and root planning (SRP). PRL was measured using enzyme‐linked immunosorbent assay.

**Results:**

At baseline, in GCF of RA + P group showed the highest mean PRL levels, followed by P group whereas groups 3 and 4 showed a statistically less values than the first 2 groups. Serum values showed non-significant difference between the first three groups, although higher than healthy controls. SRP reduced GCF and serum levels of PRL in both P groups as well as synovial fluid PRL in group 1. SRP caused no change in DAS scores while reduced ESR values were observed in group 1 after treatment.

**Conclusions:**

Local GCF and synovial levels of PRL seem to be linked to the disease process of both periodontitis and rheumatoid arthritis than serum levels. SRP reduced these local levels.

**Clinical relevance:**

In patients with RA and CP, local PRL seems to play a role in the association between the two conditions; further, periodontal treatment is essential to improve periodontal condition in RA patients.

**Trial registration:**

Clinicaltrials.gov. Identifier: NCT04279691.

## Introduction

Hormones are involved in various aspects of the immune response and rheumatic diseases; the interest in the crosstalk between hormones and cytokines acting on inflammation and bone metabolism has recently grown [1]. Rheumatoid arthritis (RA) is a chronic inflammatory autoimmune disorder mainly affecting the synovial membranes of multiple joints causing overt local proliferative inflammation, joint tissue destruction, deformities, and functional disability with a marked decline in the patient’s quality of life. Till present, the etiology of RA is not fully revealed; however, the combination of multiple risk factors, including genetic, hormonal, and environmental factors—like infections, smoking, obesity, and exposure to silica dust—is believed to contribute to the pathology. Whatever the causative factor is, hallmark of RA pathogenesis is related to the citrullination process and autoimmunity that leads to high production of the anti-citrullinated protein antibodies that form immune complexes at the synovial membrane of joints [2]. Treatment of RA targets the amelioration of inflammation and to retard the rate of joint damage by the use of conventional synthetic disease-modifying anti-rheumatic drugs (DMARDs), biological DMARDs, NSAIDs, and glucocorticoids [3].

Periodontitis (P) is another chronic inflammatory disease of teeth supporting structures, mainly induced by bacterial biofilm dysbiosis with a multifactorial contribution. Bacteria-derived factors stimulate a local inflammatory reaction, cytokines release, followed by recruitment and activation of T and B lymphocytes causing periodontal tissue destruction and teeth loss. Treatment of periodontitis aims mainly at reduction of microbial load and associated inflammation by mechanical debridement. However, due to the multifactorial nature of periodontitis, standard treatment may not be a sufficient to achieve long-term clinical improvements [4, 5].

Since the association between periodontitis and RA has been confirmed long ago by almost all the population‐based studies, now, there is a need to investigate possible mechanisms that might be responsible for such an association [6, 7]. Several clinical, epidemiological, and animal studies investigated several mechanisms underlying the association between the two conditions; however, there is still a plausibility of data regarding certain links between the two conditions like obesity and hormones expression [8].

Prolactin (PRL) is a neuroendocrine hormone that mainly regulates lactation; however, it also acts as a cytokine with both autocrine, endocrine, and paracrine effects. PRL is mainly secreted by pituitary gland besides many extra-pituitary tissues and cells. Pituitary and extra-pituitary released PRL are structurally identical and bind to the same receptors that are expressed in the pituitary as well as many other tissue cells including the heart, immune system cells, and osteoblasts. Regulation of extra-pituitary PRL is mainly site specific and independent from pituitary PRL [9]. A number of biological processes are attributed to prolactin like metabolism and immune-modulation; thus, biological effects of PRL have been linked to immune-mediated inflammatory diseases like systemic lupus, multiple sclerosis, cancer, and periodontitis. This link still needs investigations as it opens new avenues on clinical implications and consequences of hyper-prolactinemia [10].

Rahajoe et al. stated that “analysis of gingival crevicular fluid of RA patients reveals that the relationship between periodontitis and RA is bidirectional, and studying cytokines in the periodontal inflammatory exudate of RA patients might provide insight into the association between periodontitis and RA” [11]. Previously, we reported elevated levels of PRL in GCF of patients with periodontitis compared to healthy controls, and that these levels were reduced, 3 months after non-surgical periodontal therapy [10]. Consequently, we hypothesized that local prolactin expression in periodontal and synovial tissues would be elevated in patients with RA and periodontitis compared to controls [12]. This study aims to explore levels of prolactin hormone in gingival crevicular fluid, synovial fluid, and serum of patients suffering from RA and periodontitis compared to periodontitis patients, periodontally healthy RA patients, and healthy controls. Moreover, we aimed to evaluate the effect of non-surgical periodontal therapy on PRL levels in periodontitis patients with and without RA.

## Materials and methods

### Study design and ethical aspects

In this case controlled clinical trial, subjects were recruited from the outpatient clinic of the Oral Medicine and Periodontology Department, Faculty of Dentistry, October University for Modern Sciences and Arts (MSA) and Al-Azhar Universities and from Rheumatology Department, Al-Zahraa Hospital, Al-Azhar University, Cairo, Egypt, from October 2019 to December 2020. The study was conducted according to the Declaration of Helsinki (1964, revision 2008). All subjects participated voluntary and received detailed information about the study. A written consent was obtained from each participant before the start of the trial. This trial was registered on www.Clinicaltrials.gov. Identifier: NCT04279691 (21/02/2020). The research protocol was approved by the Ethics Committee of Faculty of Dentistry, MSA University ID ETH14.

### Sample size calculation

Sample size calculation was performed using G*Power version 3.1.9.2. Prolactin level in GCF after treatment was the primary outcome. A pilot study was conducted on three patients in each group (included in the study groups afterwards). Mean values were 22, 18.8, 17.5, and 18 for the four groups, respectively. Standard deviation within each group was assumed to be 4. The effect size *f* was (0.438). Using alpha (*α*) level of 5% and beta (*β*) level of 20%, i.e., power = (80%), the minimum estimated sample size was a total of 64 patients, i.e., 16 patients per group. Sample size was increased to 20 patients per group to compensate for a drop-out rate of 25%.

### Study population

A detailed medical history of each participant was obtained according to the modified Cornell Medical Index questionnaire [13], then patients underwent full dental and rheumatologic examinations. Subjects were divided into four groups with the following criteria:

#### Inclusion criteria

Group 1: patients with chronic moderately active RA [14] and stage III or IV periodontitis [15]. Group 2: patients with stage III and IV periodontitis with healthy systemic condition. Group 3: patients with chronic moderately active RA and healthy periodontium (plaque index [PI] < 1 and gingival index [GI] < 1), zero clinical attachment loss (CAL) and with no previous history of periodontal disease. Group 4: systemically and periodontally healthy subjects.

#### Exclusion criteria

(1) Pregnancy or lactation; (2) any known systemic disease other than RA for groups 1 and 3; (3) previous periodontal treatment (surgical or non-surgical) in last 6 months; (4) antibiotics in the past 3 months; (5) previous intra-articular drug injections; (6) smoking; (7) biological DMARD therapy; (8) abnormal body mass index (more than 29.9 kg/m^2^).

One rheumatologist (S.A.) did the rheumatologic examination and synovial fluid sampling and one periodontist (Z.S.) did the periodontal examination and GCF sampling; both examiners were blinded from each other for the rheumatologic, periodontal conditions and study groups. Calibration exercises for probing measurements were performed in five patients before the study, with a 0.82 k value for PD and 0.76 for CAL. Laboratory analysis was conducted by O.S. who was blinded to the study groups.

### Rheumatologic examinations

Diagnosis of RA was verified according to the 2010 RA classification criteria of the American College of Rheumatology and EULAR [16]. Active RA is defined by the presence of 12 or more tender joints, 10 or more swollen joints, and at least one of the following: erythrocyte sedimentation rate (ESR) of at least 28 mm/h, C-reactive protein level greater than 20 mg/L, or morning stiffness for at least 45 min. Patients have been taking conventional disease-modifying anti-rheumatic drugs, including methotrexate, hydroxychloroquine, and prednisone, either as monotherapy or as a combination of double therapy. Analyses of blood samples were conducted for erythrocyte sedimentation rate (ESR). Disease Activity Score (DAS) was calculated from the number of tender and swollen joints (28-joint count) and ESR [17]. All included patients had a DAS 28 ≥ 3.2 to 5.1, despite the use of disease-modifying anti-rheumatic drugs. Duration of illness and medications used by the patients were collected from the patient files and by asking the patients. DAS 28 score was used due to its accuracy; good discrimination between different disease activities; included remission criteria; and its ease for clinical implementation [18].

### Periodontal examination and treatment

Dental clinical and radiographic examinations were performed to all study subjects. All teeth were examined excluding the third molars and implants (WHO, 1997). The following measurements were recorded: (1) clinical attachment loss (CAL), (2) probing pocket depth (PD), (3) gingival index [19], and (4) plaque index [20]. Based on these periodontal outcome parameters, patients were categorized using the 2018 AAP/EFP definitions of PD cases and staging of periodontal disease [14]. Accordingly, diagnosis of periodontitis was confirmed by having PD-related detectable interdental CAL at ≥ 2 non-adjacent teeth or having a buccal or oral CAL ≥ 3 mm with pockets of > 3 mm detectable at ≥ 2 teeth. The number of stages III, IV periodontitis patients was 15, 6 and 17, 3 in groups 1 and 2, respectively. Measurements were recorded at six sites for all teeth (mesio-buccal, mid-buccal, disto-buccal, mesio-lingual, mid-lingual, and disto-lingual). After initial examination and sampling, oral hygiene instructions (including interdental cleaning) and prophylaxis sessions were performed, and subsequent evaluation of patients’ compliance was done. This was followed by full mouth scaling and root planning (SRP) for two sessions under local anesthesia, all teeth with pocket depth PD ≥ 3 mm were scaled and planed by means of sonic, ultrasonic, and hand instruments (Gracey curettes, Hu-Friedy, Chicago, IL, USA), treated pockets were then thoroughly rinsed with 0.2% chlorhexidine digluconate solution, and patients were instructed to rinse twice daily for 2 min with a 0.2% chlorhexidine digluconate for 1 week.

### Fluid sampling and examination

Synovial fluid and serum samples were collected the same day of baseline clinical examination whereas the GCF samples were collected the day after to avoid the contamination of crevicular fluid with blood associated with the probing of inflamed sites. Patients of all study groups provided serum and GCF samples at baseline; only the RA patients agreed to provide synovial fluid samples. Periodontitis patients in our study who received SRP provided GCF samples 3 months after treatment.

Synovial fluid sample collection was performed via arthrocentesis from inflamed knee joint. GCF samples were obtained from the buccal aspects of two interproximal sites in teeth that had the highest signs of inflammation and attachment loss. Selected site was first isolated with cotton rolls and air-dried and samples were collected after meticulous removal of supragingival plaque. For the periodontally healthy groups, samples were collected from the upper first molar. Filter paper strip (Periopaper; ProFlow Inc., Amityville, NY, USA) was placed in the selected sites for 30 s. Care was taken to avoid mechanical trauma and blood strips contaminated were discarded. For serum sample, 5 ml aliquot of blood was obtained. After collection, samples were immediately centrifuged and stored at − 80 °C until further use. Samples were assayed for PRL by using ELISA kits (Prechek Bio, Inc. CA, USA), according to manufacturer’s instructions using human recombinant standards. Results were expressed as concentrations in nanograms per milliliter (ng/ml); sensitivity of the kit was 5 ng/ml.

### Statistical analysis

Numerical data were explored for normality by Kolmogorov–Smirnov and Shapiro–Wilk tests. All data showed parametric distribution except for plaque index and gingival index. Data were presented as mean and standard deviation (mean ± SD) values. For parametric data, one-way ANOVA and repeated measures ANOVA tests were used to compare between the groups as well as to study the changes after treatment within each group. Bonferroni’s post hoc test was used for pair-wise comparisons when ANOVA test is significant. Paired *t*-test was used to study the changes in DAS, ESR, and prolactin level in synovial fluid after treatment in group 1. For non-parametric data, Mann–Whitney *U* test was used to compare between periodontitis groups. Wilcoxon signed-rank test was used to study the changes within each group. Qualitative data were presented as frequencies and percentages. Chi-square test was used to compare between the groups. The significance level was set at *P* ≤ 0.05. Statistical analysis was performed with IBM SPSS Statistics for Windows, version 23.0. Armonk, NY: IBM Corp.

## Results

### Baseline characteristics

Eighty participants including 36 males and 44 females, age range from 18 to 75 years, were tested. All groups had more females except for healthy control group, with non-significant difference. In groups 1 and 3, mean value (mean ± SD) for RA disease duration was 44 ± 21.2 and 27.5 ± 10.8 months, respectively. Mean values (mean ± SD) for DAS were 4.2 ± 0.6 and 4 ± 0.7 in groups 1 and 3, while mean values (mean ± SD) for ESR were 29.4 ± 3.3 and 29 ± 2.1, respectively. At baseline, 40% of RA patients in group 1 were on monotherapy and 60% had double therapy; after 3 months, 40% were using monotherapy and 50% used double therapy and 10% were on triple therapy. In group 3, 40% of RA patients had monotherapy and 60% had double therapy. In groups 1 and 2, periodontitis grades III and IV showed equal distribution (50% for each grade) (Table 1).

**Table 1:** Mean, standard deviation (SD), frequencies (*n*), percentages, results of one-way ANOVA test, Kruskal–Wallis test, and chi-square test for comparison between baseline characteristics in the four groups

	RA and periodontitis (*n* = 20)	Chronic periodontitis (*n* = 20)	RA without periodontitis (*n* = 20)	Control (*n* = 20)	*P* value
Age (years) (mean ± SD)	60.9 (6.9)^A^	53.1 (5.3)^A^	27.5 (10.5)^C^	39.1 (9.4)^B^	< 0.001*
Gender [*n* (%)]					0.489
Male	8 (40%)	8 (40%)	8 (40%)	12 (60%)	
Female	12 (60%)	12 (60%)	12 (60%)	8 (40%)	
RA treatment [*n* (%)]					1
Monotherapy	8 (40%)	–	8 (40%)	–	
Double therapy	12 (60%)		12 (60%)		
DAS (mean ± SD)	4.2 (0.6)		4 (0.7)		0.941
ESR (mean ± SD)	29.4 (3.3)		29 (2.1)		0.887*
PI (mean ± SD)	3.5 (0.51)	2.3 (0.47)	–	–	< 0.001*
GI (mean ± SD)	2.7 (0.47)	2.7 (0.47)	–	–	1
PD (mm) (mean ± SD)	7 (0.79)	6.3 (0.8)	–	–	0.009*
CAL (mm) (mean ± SD)	7.2 (1.01)	8.8 (1.44)	–	–	< 0.001*

^*^Significant at *P* ≤ 0.05. Different superscripts indicate statistically significant difference between groups

### Prolactin levels before and after non-surgical periodontal therapy

For GCF, at baseline, RA + P group showed the highest mean prolactin levels, followed by periodontitis group that showed a significantly lower mean level while RA without periodontitis and control groups showed the lowest mean prolactin levels (*P* value < 0.001, effect size = 0.911). After treatment, a significant reduction was reported in groups 1 and 2 compared to pre-treatment levels (*P* value < 0.001, effect size = 0.789 and *P* value < 0.001, effect size = 0.712), with a non-significant difference between both groups. Groups 1 and 2 showed significantly higher mean PRL levels than groups 3 and 4 after treatment (Table 2).

**Table 2:** Descriptive statistics and results of repeated measures ANOVA test for comparison between prolactin levels in the three groups, changes by time within each group and paired *t*-test for comparison between prolactin levels in synovial fluid before and after treatment

Site	Time	RA and periodontitis (*n* = 20)		Chronic periodontitis (*n* = 20)		RA without periodontitis (*n* = 20)		Control (*n* = 20)		*P* value	Effect size (partial eta squared)
		Mean	± SD	Mean	± SD	Mean	± SD	Mean	± SD		
GCF	Before treatment	39.8^A^	5.4	35.1^B^	5.2	13.6^C^	1.9	12.6^C^	1.5	< 0.001*	0.911
	After treatment	25^A^	4.7	23^A^	3.7	13.6^B^	1.9	12.6^B^	1.5	< 0.001*	0.755
*P* value (effect of time)		< 0.001*		< 0.001*		–		–			
Effect size (partial eta squared)		0.789		0.712		–		–			
Serum	Before SRP	38.8^A^	5.1	35.7^A^	4.4	36.5^A^	6.9	29.7^B^	3.4	< 0.001*	0.309
	After SRP	35.6^A^	6.4	33.9^A^	4.6	36.5^A^	6.9	29.7^B^	3.4	< 0.001*	0.189
*P* value (effect of time)		< 0.001*		< 0.008*		–		–			
Effect size (partial eta squared)		0.292		0.119		–		–			
SF	Before SRP	44.9	5.5	–		44.7	6.2	–		0.919	0.0003
	After SRP	41.9	4	–				–			
*P* value (effect of time)		0.001*		–		–		–			
Effect size (*d*)		0.834		–		–		–			

^*^Significant at *P* ≤ 0.05. Different superscripts in the same row indicate statistically significant difference between groups. *GCF*, gingival crevicular fluid; *SF*, synovial fluid

For serum PRL, at baseline, a non-significant difference between groups 1, 2, and 3 was found. The 3 groups (1, 2, and 3) showed significantly higher mean PRL levels than control group. After treatment, the two intervention groups reported a significant decrease in serum PRL compared to pre-treatment, with a non-significant difference. Serum PRL in groups 1, 2, and 3 remained significantly higher than the control group. At baseline, synovial fluid PRL levels in RA with periodontitis patients were non-significantly higher than RA patients without periodontitis. Within group 1, PRL levels were significantly decreased after treatment compared to pretreatment levels (*P* value = 0.001, effect size = 0.834) (Table 2).

### Clinical parameters

For RA parameters, a non-significant change in DAS scores after treatment was reported; mean values (mean ± SD) were 4.2 ± 0.6 and 4.1 ± 0.6 pre- and post-treatment, respectively. The mean values (mean ± SD) for ESR were 29.4 ± 3.3 and 28.1 ± 3.3 pre- and post-treatment, with a significant decrease (Table 3).

**Table 3:** Descriptive statistics and results of Mann–Whitney *U* test, Wilcoxon signed-rank test, and repeated measures ANOVA test for comparison between clinical parameters in periodontitis groups and changes by time within each group

Clinical parameters	Time	RA and periodontitis (*n* = 20)		Chronic periodontitis (*n* = 20)		*P* value	Effect size
		Mean	SD	Mean	SD		
PI	Before treatment	3.5	0.51	2.3	0.47	< 0.001*	*d* = 2.118
	After treatment	2.3	0.47	1.2	0.89	< 0.001*	*d* = 1.462
*P* value (effect of time)		0.006*		0.027*			
Effect size (*d*)		1.571		1.136			
GI	Before treatment	2.7	0.47	2.7	0.47	1	*d* = 0
	After treatment	1.2	0.89	0.3	0.47	< 0.001*	*d* = 2.327
*P* value (effect of time)		0.010*		0.004*			
Effect size (*d*)		1.404		1.663			
PD (mm)	Before treatment	7	0.79	6.3	0.8	0.009*	Partial eta squared = 0.168
	After treatment	3.5	0.51	3.3	0.66	0.290	Partial eta squared = 0.029
*P* value (effect of time)		< 0.001*		< 0.001*			
Effect size (partial eta squared)		0.935		0.914			
CAL (mm)	Before treatment	8.8	1.44	7.2	1.01	< 0.001*	Partial eta squared = 0.305
	After treatment	4.9	1.07	4.2	0.77	0.023*	Partial eta squared = 0.129
*P* value (effect of time)		< 0.001*		< 0.001*			
Effect size (partial eta squared)		0.933		0.892			

*Significant at *P* ≤ 0.05. *PI*, plaque index; *GI*, gingival index; *PD*, pocket depth; *CAL*, clinical attachment loss

For periodontal parameters at baseline, RA + P group showed statistically significantly higher mean PD, CAL, and PI records compared to P group—except for GI—that showed a non-significant difference. After treatment, groups 1 and 2 reported a significant decrease in all recorded parameters compared to pretreatment levels within each group. For post-treatment records comparison, group 1 reported a significant increase compared to group 2 (except for the PD values that showed a non-significant difference between the 2 groups). Post-treatment values of groups 1 and 2 remained significantly higher than group 3 (Table 3).

## Discussion

The present study reports, for the first time, gingival crevicular fluid, synovial fluid, and serum levels of prolactin hormone in RA patients with and without periodontitis and the effect of non-surgical periodontal therapy on these levels. We included a well distinguished group of RA patients with moderate severity, not consuming biological DMARDs and those with periodontitis had equal affection between stages III and IV. The RA + P patients had significantly worsened periodontal condition at baseline compared to periodontitis group patients and healthy controls. This is consistent with previous data that reported the same findings [3, 21]. The deteriorated periodontal condition in RA patients was attributed to the elevated systemic inflammatory burden that enhances the severity of periodontal inflammation; further, the limited manual dexterity in RA patients causes difficulties in maintaining satisfactory oral hygiene [22]. However, other studies reported no differences in periodontal condition between RA patients and controls [23, 24]; this discrepancy could be explained by difference in study design, tested population, the severity of RA condition, medications taken etc.

In this work, RA patients treated with the biologic DMARDs were excluded to avoid its possible effect on the inflammatory status and hence, PRL expression. Earlier studies reported that biologic DMARDs ameliorate periodontal inflammation in patients with RA and increase patient’s susceptibility to infection [5, 22]. Our study included patients undergoing mono or double therapy drug combinations, as it was earlier reported that RA medication (either mono, double, or triple therapy) did not seem to differently affect the severity of periodontal status [25].

The non-surgical periodontal therapy caused significant reduction in periodontal parameters in this study, supporting earlier data that reported improvement of the periodontal condition after treatment in both RA + P and P groups [26–28]. Our data concordat with that reported by Cosgarea et al., as the periodontal treatment did not cause a significant reduction in DAS scores, but caused a significant reduction in ESR [28]. Other studies reported a significant reduction in DAS 28, 3–6 months after SRP [26, 29]. Reduction in ESR after SRP was reported by Erciyas et al. [29], where others reported no change in ESR after treatment [27, 30]. A variety of factors can explain this discrepancy including baseline criteria of the RA patients, type of medication used, RA disease activity, sample size, and methodological differences [27].

The concept of the locally produced PRL at sites of tissue pathosis via activating its receptor on target cells has emerged as a new mechanism in various pathologic contexts, including inflammation and cancer [30–32]. In this work, local PRL levels were higher in crevicular and synovial fluids of PRL in RA + P patients compared to periodontitis patients and both were greater compared to non-periodontitis RA patients and healthy controls. Our study lacks data regarding the PRL synovial levels in periodontitis and healthy control groups. Arthrocentesis procedure is considered by most of the patients to be painful and invasive; over 80% of the recruited patients in these groups refused to provide synovial fluid samples upon explanation of the study aim and methodology.

The high GCF expression of PRL in periodontitis patients with and without RA and its reduction after SRP agrees with earlier reports in which the local expression of prolactin increases with active disease process and reduced significantly after treatment; this further supports the value of this hormone in the inflammatory process associated with periodontitis [10, 31]. Post-treatment levels were higher than healthy controls, indicating the chronicity of periodontitis in its patients [33]. The elevated crevicular expression of PRL in periodontitis can be attributed to mediators for periodontal tissue destruction like IL-1 and TNF-alpha that are well known stimulators of PRL secretion; however, this needs to be supported by further in vivo and in vitro studies to prove this explanation [34].

The pronounced synovial PRL expression in our RA patient agrees with Tang et al. findings; they attributed this to possible partial vascular leakage of the pituitary originated hormone into synovial tissue and from peripheral monocytes capable of PRL production, PRL and its receptors were suggested to be linked to RA pathology by local crosstalk (either by autocrine or paracrine ways), between the immune and endocrine systems [32, 35]. Further supporting our findings, elevated PRL local production by synovial T cells, fibroblasts, macrophages, and cells markedly expressing PRLR was reported in RA patients [36, 37]. In our study, the synovial PRL in RA + P patients were higher compared to RA patients without periodontitis. This is easily interrupted by the systemic status of chronic sub-clinical inflammation associated with periodontitis [38]. Targeting locally produced PRL with its receptor in synovial tissues of RA patients as a potential therapeutic strategy for reducing signs of joint inflammation has been suggested and tested. Systemic bromocriptine ingestion improved signs and symptoms of RA patients [32, 39], although others failed to confirm this finding [4].

High PRL serum levels in patients with immune-inflammatory conditions like RA, psoriatic arthritis, multiple sclerosis, and systemic lupus were reported, highlighting the relation between PRL and these diseases [1, 12]. In RA, conflicting data regarding serum PRL levels in patients with RA compared to controls were reported; some reported a significant increase [40], while others reported a no difference [35, 41]. In this work, no difference was reported at baseline between the three diseased groups and all were higher than healthy controls; further, in periodontitis groups (with and without RA), SRP did not significantly reduce serum PRL levels after treatment. Our findings suggest a minor role of systemic PRL in pathogenesis of RA and periodontitis as serum levels could be influenced by a variety of factors like obesity and plasma insulin levels [42].

### Strengths and limitations

The weakness in our study is the rather unusual study design, but we had many questions to answer. In general, non-randomized controlled studies are more susceptible to bias; however, this is partially counterbalanced by the inclusion of a well characterized group of patients (RA patient with moderate activity and stage III or IV periodontitis patients) to ensure quality of reporting. Another limitation is the lack of synovial fluid samples in periodontitis patients without RA and healthy controls.

Finally, our results suggest a role for prolactin in the pathogenesis of both RA and P, and a positive effect of non-surgical periodontal treatment in management of periodontal inflammation in RA patients. However, the underlying mechanisms for the pronounced expression of PRL in periodontal and synovial tissues, and in different stages of disease development are still lacking. Also, the possible confounders that affect the activity of PRL such as cell type, PRL concentration, stimulus duration, PRLR isoform, and cytokines in the medium need to be investigated.
